# Genetically high angiotensin-converting enzyme concentrations causally increase asthma risk: A meta-analysis using Mendelian randomization

**DOI:** 10.3389/fmed.2022.941944

**Published:** 2022-11-07

**Authors:** Qin Hui, Ying Hao, Fang Ye, Bo Pang, Wenquan Niu, Qi Zhang

**Affiliations:** ^1^Department of Pediatrics, China-Japan Friendship Hospital, Beijing, China; ^2^Institute of Clinical Medical Sciences, China-Japan Friendship Hospital, Beijing, China

**Keywords:** angiotensin-converting enzyme, asthma, meta-analysis, polymorphism, Mendelian randomization

## Abstract

**Objectives:**

This meta-analysis aimed to test the association of angiotensin-converting enzyme (*ACE*) gene I/D polymorphism with asthma risk and circulating ACE changes.

**Methods:**

Public literature retrieval, publication selection, and information extraction were completed independently by two investigators. Effect-size values are expressed as odds ratios (ORs) or standardized mean differences (SMDs) with a 95% confidence interval (95% CI).

**Results:**

Nineteen studies (2,888 patients and 9,549 controls) fulfilled the eligibility criteria. Overall investigations demonstrated that *ACE* gene I/D polymorphism was significantly associated with asthma risk under allelic (OR, 95% CI: 1.26, 1.08 to 1.48), homozygous genotypic (1.50, 1.09 to 2.06), and recessive (1.53, 1.24 to 1.89) models with moderate heterogeneity (*I*^2^ statistic: 64% to 79%). Subsidiary investigations recorded that race, matched status, asthma diagnosis, sample size, and age possibly accounted for the existence of significant heterogeneity. Relative to carriers with the II genotype, those with the DD genotype, ID genotype, and the combination of DD and ID genotypes had significantly higher concentrations of circulating ACE (WMD: 3.13, 2.07, and 2.83 U/L, respectively, *p* < 0.05). Adoption of Mendelian randomization analyses revealed that one unit increment in circulating ACE concentrations was found to be significantly associated with a 1.14-fold increased risk of asthma (95% CI: 1.02 to 4.24).

**Conclusion:**

We provided strong meta-analytical evidence supporting the causal implication of high circulating ACE concentrations in the development of asthma.

## Introduction

Asthma is a highly heritable disease. The heritability of childhood asthma reached as high as 82% (1). A long list of asthma-susceptibility genes has been identified (2, 3). However, no consensus exists on which gene actually involves the pathogenesis of asthma, even with genome-wide association studies (4–6). In this regard, the candidate gene approach still represents an alternative strategy (7). Based on a known biological function, a gene can be a candidate to precipitate asthma. Importantly, the gene can be screened to see which mutation actually embodies its function. One such case is the insertion/deletion (I/D) polymorphism in the gene coding angiotensin-converting enzyme (ACE).

The association of *ACE* gene I/D polymorphism with asthma has been widely studied. For example, the DD genotype of the *ACE* gene was overrepresented in patients with asthma relative to healthy controls (8). In an early meta-analysis, the DD homozygote carriers had an overall about 60% increased risk of asthma compared with the II + ID carriers, and this risk was more evident in Asians (9). A recent meta-analysis in children indicated that *ACE* gene I/D polymorphism was associated with a significant risk of asthma (10). Other studies, however, did not reveal any significance between this polymorphism and asthma risk (11–13). The reasons behind this inconsistency are multiple, likely because of differences in origins, baseline characteristics of study participants, diagnosis criteria of asthma, and statistical power to derive significance. To this point, the synthesis of individually underpowered studies with the same research goals can help shed some light on these reasons.

To yield robust evidence, we aimed to perform an updated meta-analysis and test the association of *ACE* gene I/D polymorphism with asthma risk and changes in circulating ACE concentrations. Meanwhile, heterogeneity sources attributable to inconsistent observations were explored.

## Methods

### Performance guidelines

**Figure 1:**
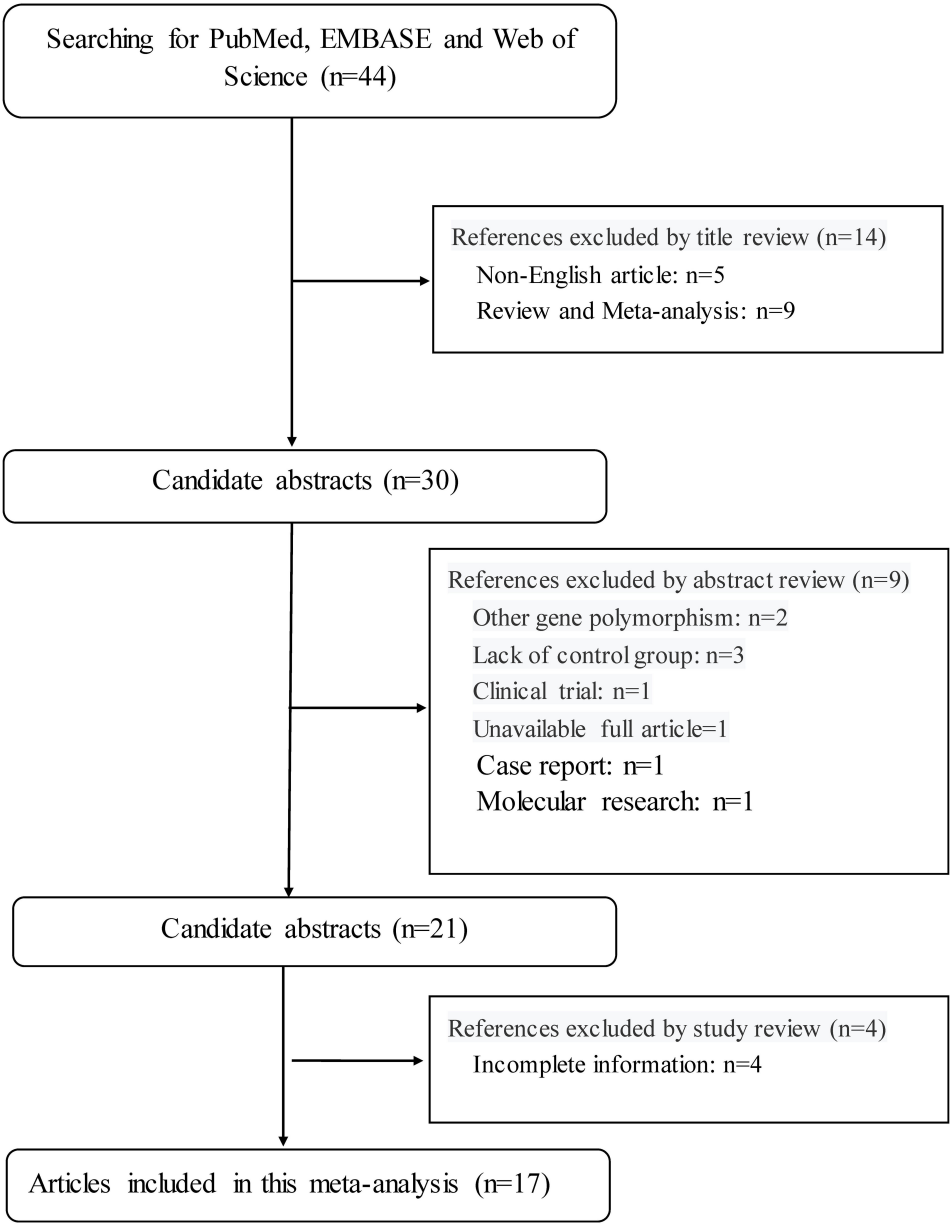
The PRISMA flowchart illustrates the selection process of qualified articles in this meta-analysis.

This meta-analysis was performed according to the guidelines in the preferred reporting items for systematic reviews and meta-analyses (PRISMA) statement. The PRISMA checklist is shown in Supplementary Table 1, and the PRISMA flow diagram is shown in Figure 1.

### Search strategies

Using predefined key terms, four electronic databases—PubMed, HuGE Navigator, EMBASE (Excerpt Medica Database), and Web of Science—were searched from literature inception through March 2022. Key terms used for searching possibly eligible publications were formulated using the MeSH (Medical Subject Headings) database and are expressed in the Boolean format, that is (“asthma” or “atopic”) and (“polymorphism” or “SNP” or “variant” or “mutation” or “variation” or “genetic” or “genotype” or “allele”) and (“ACE” or “angiotensin-converting enzyme” or “angiotensin I converting enzyme” or “ACE1” or “DCP” or “DCP1” or “CD143”).

Initial screening of searched publications was restricted to the English language and human beings. The search was also extended to the reference lists of major publications (reviews and meta-analyses) retrieved. The search was independently performed by two investigators (Q. H. and F. Y.), and any difference in numbers was resolved *via* discussion and a consensus was attained.

### Inclusion criteria

Included publications must concurrently meet the following four criteria: (i) class of evidence: case-control design; (ii) outcome: asthma with clear definition; (iii) necessary data: genotype counts (or allele counts in the absence of genotype counts) of *ACE* gene I/D polymorphism between patients with asthma and controls, or circulating ACE concentrations across I/D genotypes in either patients or controls or both; and (iv) genotype determination: valid methodology.

### Exclusion criteria

Publications were excluded if one or more of the following criteria were met: (i) type of publication: review, letter to editor or correspondence, editorial, comment, conference abstract, and case report or series; (ii) publication with duplicate participant samples; (iii) involvement of only cases; (iv) endpoint other than asthma; and (v) unpublished data.

### Publication selection

The selection of eligible publications was handled in two steps. First, the title and abstract (if available) were reviewed and removal was applied based on exclusion criteria. After the first round of publication removal, the full text was read and eligibility was checked based on inclusion criteria.

The selection process of eligible publications was completed independently by two investigators (Q. H. and F. Y.), and any divergence was solved by discussion and if necessary by a third investigator (W. N.).

### Information extraction

By the use of a uniform data extraction Excel sheet, information from each qualified publication was independently extracted by two investigators (Q. H. and F. Y.), and two Excel sheets were compared by kappa statistics. Any disagreement was solved by rechecking the full text until a consensus was attained.

The first author's name, year of publication, country where participants were enrolled, race, sample size, design, source of controls, matched condition, diagnosis of asthma, genotype counts of *ACE* gene I/D polymorphism, and baseline characteristics of study participants, including age, gender, and ACE concentrations in circulation, were all extracted.

### Statistical analyses

STATA software version 14.1 for Windows (Stata Corp, College Station, Texas) was utilized for this meta-analysis. The association of *ACE* gene I/D polymorphism with the risk of asthma was measured by odds ratio (OR) with a 95% confidence interval (95% CI). Changes in circulating ACE concentrations between genotypes of this polymorphism were denoted by standardized mean difference (SMD) with a 95% CI. Both OR and SMD were derived under the random-effects model, because in the absence of heterogeneity, fixed-effects and random-effects models yield very similar estimates, whereas, in the presence of heterogeneity, the random-effects model is preferred (14). In the case of the significant association of *ACE* gene I/D polymorphism with asthma risk and ACE changes in circulation, the Mendelian randomization technique was employed to infer the possible causality between circulating ACE and asthma.

Between-study heterogeneity was measured by the inconsistency index (*I*^2^) statistic. *I*^2^ denotes the percent of variability observed between studies that are the result of heterogeneity but not a chance observation. Higher *I*^2^ indicates a higher likelihood of heterogeneity. An *I*^2^ statistic of over 50% is indicative of significant heterogeneity. The sources of heterogeneity were statistical, clinical, and methodological aspects. To track these sources, subsidiary analyses according to pre-specified factors (including sample size, race, matched condition, source of controls, and diagnosis of asthma) and meta-regression analyses of both continuous and categorical factors were carried out. Subgroups involving two or more studies are displayed.

In addition, cumulative analyses were conducted to see the impact of the first publication on subsequent publications and evolution of accumulated estimates over time. Sensitivity analyses were conducted to see the impact of any single study on the overall effect-size estimate by removing an individual study each time to check whether any of these estimates can bias the overall estimates.

Publication bias was assessed by Begg's funnel plot. If the funnel shape is asymmetrically inverted, it suggests a correlation between pooled estimate and study size (publication bias or small study bias). From a statistical aspect, publication bias was measured by Egger's test, which appraises funnel asymmetry with a significance level set at 10%.

## Results

### Qualified publications

By the use of key terms, searching four public databases yielded 44 publications in the English language. Application of inclusion criteria and exclusion criteria, 17 of 44 publications were qualified for synthesis in this meta-analysis (8, 12, 13, 15–28).

In total, 19 independent studies were isolated from 17 qualified publications, including 2,888 patients with asthma and 9,549 controls, among who the genotypes of *ACE* gene I/D polymorphism were assayed with validated typing methods. Out of 19 independent studies, 3 (including 431 subjects) provided data on the changes in circulating ACE concentrations across the genotypes of this polymorphism.

### Baseline characteristics

The baseline characteristics of qualified studies are shown in Table 1. Thirteen studies were performed among adults, three among children, and one among both.

**Table 1:** Study characteristics from all qualified publications in this meta-analysis.

**Author**	**Year**	**Object**	**Country**	**Race**	**Design**	**Diagnosis**	**Source**	**Control feature**
Benessiano	1997	Adults	France	Caucasians	Cross-sectional	ATS	Hospital	Healthy
Tomita	1998	Adults	Japan	East Asians	Cross-sectional	ATS	Population	Healthy
Holla	1998	Adults	Czech	Caucasians	Cross-sectional	ATS	Hospital	Healthy
Gao	1998	Adults	UK	Caucasians	Cross-sectional	Doctor	Hospital	Healthy
Gao	1998	Adults	Japan	East Asians	Cross-sectional	Doctor	Hospital	Healthy
Gao	1998	Children	Japan	East Asians	Cross-sectional	Doctor	Hospital	Healthy
Chagani	1999	Adults	Canada	Caucasians	Cross-sectional	Doctor	Hospital	Healthy
Nakahama	1999	Adults	Japan	East Asians	Cross-sectional	ATS	Hospital	Non-asthma
Gao	2000	Adults	China	East Asians	Cross-sectional	ATS	Hospital	Healthy
Lee	2000	Adults	Korea	East Asians	Cross-sectional	ATS	Hospital	Healthy
Yildiz	2004	Adults	Turkey	Middle Eastern	Cross-sectional	ATS	Hospital	Healthy
Urhan	2004	Adults	Turkey	Middle Eastern	Cross-sectional	ATS	Hospital	Healthy
Lue	2006	Children	Taiwan	East Asians	Cross-sectional	ATS	Hospital	Non-asthma
Eryuksel	2009	Adults	Turkey	Middle Eastern	Cross-sectional	ATS	Hospital	Healthy
Lee	2009	Adults	Denmark	Caucasians	Cross-sectional	Self-report	Hospital	Non-asthma
Guo	2009	Children	China	East Asians	Prospective	GINA	Hospital	Non-asthma
Shafei	2011	Adults	Egypt	Middle Eastern	Cross-sectional	GINA	Hospital	Healthy
Bora	2013	Children	Turkey	Middle Eastern	Cross-sectional	Doctor	Hospital	Non-asthma
Saba	2016	Adults	Pakistan	Middle Eastern	Cross-sectional	Doctor	Hospital	Healthy
**Sample size**		**Allele in cases**		**Allele in controls**		**Genotypes in cases**			**Genotypes in controls**		
**Case**	**Control**	**D**	**I**	**D**	**I**	**DD**	**ID**	**II**	**DD**	**ID**	**II**
79	54	104	54	61	47	37	30	12	15	31	8
71	142	55	87	101	183	9	37	25	16	69	57
161	141	169	111	133	149	52	65	23	29	75	37
150	150	174	126	167	133	55	64	31	48	71	31
200	100	171	229	98	102	42	87	71	25	48	27
100	100	95	105	98	102	27	41	32	25	48	27
224	252	258	204	274	224	79	100	52	72	130	47
119	208	94	144	152	264	22	50	47	28	96	84
50	50	61	39	42	58	23	15	12	8	26	16
167	121	244	376	96	146	43	158	109	23	50	48
49	49	47	37	46	46	15	17	10	13	20	13
100	88	108	92	84	92	30	48	22	14	56	18
105	102	74	136	50	154	17	40	48	4	42	56
97	96	122	72	84	108	39	44	14	18	48	30
602	7,079	646	574	8,587	8,261	166	314	130	2,208	4,171	2,045
149	165	178	120	122	208	71	36	42	41	40	84
30	30	40	20	32	28	14	12	4	10	12	8
102	101	120	84	104	98	32	56	14	22	60	19
333	521	328	338	600	442	94	140	99	137	326	58

### Overall analyses

**Figure 2:**
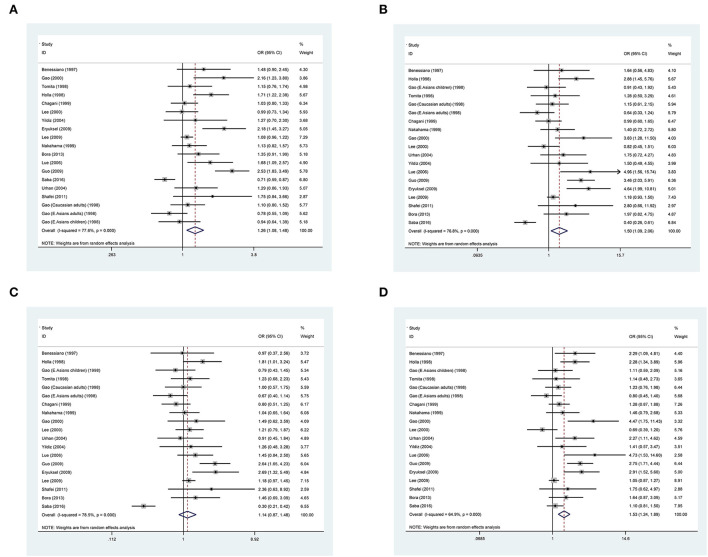
Forest plots of the angiotensin-converting enzyme (ACE) gene insertion/deletion (I/D) polymorphism in association with asthma under allelic **(A)**, homozygous genotypic **(B)**, dominant models **(C)**, and recessive model **(D)**.

The association between *ACE* gene I/D polymorphism and asthma risk was separately evaluated under allelic, homozygous genotypic, dominant, and recessive models of inheritance. Figure 2 shows the overall forest plots of the four models. This polymorphism was significantly associated with asthma risk in the allelic model, with the D allele corresponding to 1.26 times more likely to have asthma than the I allele (95% CI: 1.08 to 1.48). Significance was also noticed under homozygous genotypic (DD vs. II: OR = 1.50, 95% CI: 1.09 to 2.06) and recessive (DD vs. ID plus II: OR = 1.53, 95% CI: 1.24 to 1.89) models. However, there was no observable association under the dominant model (DD plus DI vs. II: OR = 1.14, 95% CI: 0.87 to 1.48). As of between-study heterogeneity, the *I*^2^ statistic ranged from 64 to 79% across the four models of inheritance, suggesting that diversity in effect-size estimates was not due to chance.

In addition, this association was also evaluated under the heterozygous model of inheritance (Supplementary Figure 1), and there was no hint of statistical significance (D/I vs. II: OR = 0.96, 95% CI: 0.74 to 1.25). Considering the opposite effects of allele D and allele I on asthma risk, the heterozygous model is only interrogated in overall analyses.

### Cumulative and influential analyses

Supplementary Figures 2, 3 exhibit the cumulative and influential analyses on the association between *ACE* gene I/D polymorphism and asthma risk, respectively. The cumulative impact over time was stabilized since the year 2000. Influential analyses were conducted by evaluating the impact of each study on the pooled OR *via* the deletion of one study each time, which revealed no single study impacted the pooled ORs significantly.

### Publication bias

**Figure 3:**
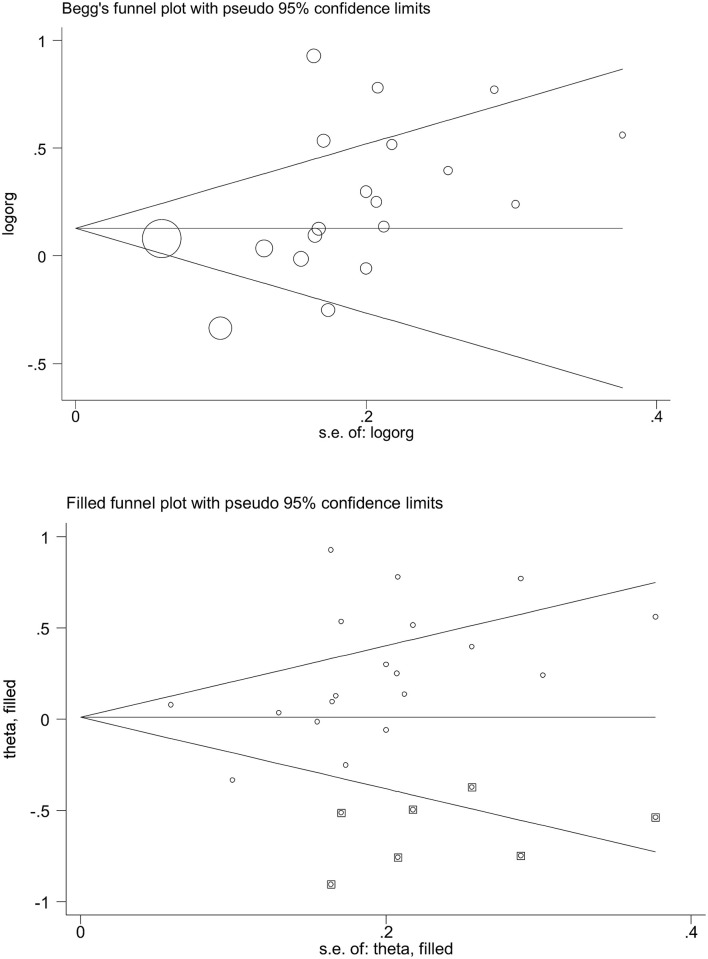
Begg's and filled funnel plots of the angiotensin-converting enzyme (ACE) gene insertion/deletion (I/D) polymorphism in association with asthma under the allelic model.

Figure 3 shows Begg's funnel plot inspecting the potential satisfaction of publication bias under the allelic model. The Begg's funnel plot seemed asymmetrical by inspection, which was confirmed by Egger's test, with the probability being 0.052.

### Subgroup analyses

As summarized in Table 2, the association between *ACE* gene I/D polymorphism and asthma risk was examined upon stratification by several potential factors on a categorical scale under the four models of inheritance.

**Table 2:** Subgroup analyses of *ACE* gene I/D polymorphisms in association with asthma.

**Subgroups**	**Studies**	**Allele model (D vs. I)**					**Homozygous genotype model (DD vs. II)**					**Dominant model (DD**+**DI vs. II)**					**Recessive model (DD vs. DI**+**II)**				
	**(cases/ controls)**	**OR**	**LCI**	**UCI**	***p***	***I*^2^ (%)**	**OR**	**LCI**	**UCI**	***p***	***I*^2^ (%)**	**OR**	**LCI**	**UCI**	***p***	***I*^2^ (%)**	**OR**	**LCI**	**UCI**	***p***	***I*^2^ (%)**
**By race**																					
Caucasians	1,216/7,676	1.19	1.00	1.41	0.05	49.7	1.32	0.96	1.82	0.08	42.5	1.12	0.89	1.41	0.34	24.6	1.41	1.04	1.90	0.03	62.1
East Asians	961/988	1.29	0.96	1.74	0.10	80.6	1.56	0.91	2.66	0.10	75.2	1.21	0.89	1.66	0.22	61.8	1.55	0.96	2.52	0.08	75.8
Middle Eastern	711/885	1.31	0.87	1.98	0.20	84.3	1.66	0.65	4.21	0.29	86.5	1.15	0.49	2.73	0.75	88.7	1.67	1.17	2.38	0.01	45.6
**By design**																					
Cross-sectional	2,739/9,384	1.19	1.04	1.38	0.01	69.3	1.4	1.03	1.91	0.03	72.70	1.07	0.83	1.38	0.60	75.20	1.45	1.18	1.78	0.00	59.00
Prospective	149/165	2.53	1.83	3.49	0.00	NA	3.46	2.03	5.91	0.00	NA	2.64	1.65	4.23	0.00	NA	2.75	1.71	4.44	0.00	NA
**By matched status**																					
Yes	707/979	1.34	0.77	2.32	0.30	91.7	1.62	0.53	4.93	0.39	91.50	1.11	0.44	2.81	0.83	93.50	1.76	1.02	3.03	0.04	72.30
No	2,181/8,570	1.22	1.06	1.41	0.01	58.8	1.43	1.09	1.88	0.01	55.80	1.12	0.94	1.34	0.19	30.50	1.47	1.16	1.87	0.00	63.20
**By diagnosis**																					
ATS	998/1,051	1.42	1.12	1.69	0.00	46.0	1.99	1.34	2.96	0.00	52.30	1.31	1.08	1.60	0.01	0.00	1.92	1.33	2.79	0.00	61.60
GINA	179/195	2.39	1.76	3.2	0.00	0.0	3.38	2.05	5.57	0.00	0.00	2.61	1.68	4.06	0.00	0.00	2.55	1.65	3.93	0.00	0.00
Doctor	1,109/1,224	0.94	0.77	1.15	0.55	60.8	0.85	0.54	1.34	0.48	69.90	0.72	0.45	1.17	0.19	80.70	1.16	0.97	1.39	0.12	0.00
Self-report	602/7,079	1.08	0.964	1.22	0.18	NA	1.18	0.93	1.5	0.17	NA	1.18	0.97	1.45	0.10	NA	1.05	0.88	1.27	0.59	NA
**By sample size**																					
>250	2,105/8,737	1.13	0.91	1.41	0.28	85.5	1.39	0.99	1.99	0.06	80.40	1.10	0.82	1.48	0.52	82.90	1.43	1.15	1.79	0.00	66.60
<250	783/812	1.44	1.21	1.71	0.00	30.4	2.21	1.24	3.94	0.01	0.00	1.38	0.82	2.24	0.23	0.00	2.25	1.41	3.59	0.00	9.60
**By age**																					
Children	456/468	1.54	0.99	2.37	0.05	80.7	2.28	1.11	4.69	0.03	69.90	1.48	0.87	2.51	0.15	69.40	2.04	1.19	3.48	0.01	60.90
Adults	2,432/9,081	1.19	1.01	1.39	0.03	71.9	1.33	0.95	1.86	0.09	74.10	1.06	0.79	1.42	0.72	78.50	1.41	1.14	1.76	0.00	60.30

ACE, angiotensin-converting enzyme; OR, odds ratio; LCI, 95% lower confidence interval; UCI, 95% upper confidence interval; I^2^, inconsistency index; NA, not available.

It is worth noticing that race, matched status, asthma diagnosis, sample size, and age were possible sources of between-study heterogeneity, particularly under the recessive model. For example, the mutation of *ACE* gene I/D polymorphism was associated with the significant risk of asthma in Caucasians under allelic and recessive models, with the odds reaching 1.19 (95% CI: 1.00 to 1.41) and 1.41 (95% CI: 1.04 to 1.90), respectively, and no significance was observed in East Asians, irrespective of the models of inheritance.

The majority of subgroups showed improved between-study heterogeneity by pooling studies with homogeneous characteristics of interest.

### Meta-regression analyses

An alternative way to explore sources of between-study heterogeneity is to perform meta-regression analyses. By regressing age, gender, asthma severity, race, matched status, asthma diagnosis, sample size, and study design (Supplementary Table 2), no hints of significance were seen at a significance level of 5%.

### Genotype–phenotype analyses

The relationship between *ACE* gene I/D polymorphism and circulating ACE concentrations is shown in Table 3. Relative to carriers with the II genotype, those with the DD genotype, ID genotype, and the combined DD and ID genotypes showed significantly higher concentrations of circulating ACE (WMD: 3.13, 2.07, and 2.83 U/L, respectively, *p* < 0.05 for all).

**Table 3:** Mean changes in circulating ACE concentrations between carriers of different genotypes of *ACE* gene I/D polymorphism.

**Models**	**Studies**	**WMD**	**95% LCI**	**95% UCI**	***p***	***I*^2^ (%)**	**Egger's *P*-value**
DD vs. II	3	3.13	0.50	5.77	0.02	96	0.59
ID vs. II	3	2.07	0.33	3.81	0.02	96	0.42
DD+ID vs. II	3	2.83	0.38	5.28	0.02	97.9	0.63

ACE, angiotensin-converting enzyme; WMD, weighted mean difference; 95% LCI, 95% lower confidence interval; 95% UCI, 95% upper confidence interval; I^2^, inconsistency index.

### Mendelian randomization analyses

Given the significance observed in both genotype-disease and genotype–phenotype analyses, the Mendelian randomization technique was utilized to infer the possible causal association between circulating ACE and asthma risk. Under the assumptions of the Mendelian randomization technique and by use of *ACE* gene I/D polymorphism as an instrumental variable, one unit increment in circulating ACE concentrations was found to be significantly associated with a 1.14-fold increased risk of asthma (95% CI: 1.02 to 4.24) under the homozygous genotypic model.

## Discussion

The aim of this meta-analysis was to test the association of *ACE* gene I/D polymorphism with asthma risk and circulating ACE changes as well as explore sources for heterogeneity in the English literature. Through a comprehensive pooling of 19 independent studies involving 2,888 cases and 9,549 controls, this polymorphism was associated with a significant risk of asthma and changes in circulating ACE concentrations. Importantly, further adoption of the Mendelian randomization technique revealed that genetically increased ACE concentrations were causally associated with an increased risk of asthma. To the best of our knowledge, this is thus far the first meta-analytical evidence concerning the causal relation between circulating ACE and asthma risk in the literature.

It is well-known that asthma is a chronic pulmonary disease characterized by intermittent and reversible airflow obstruction (29). The exact etiology of asthma currently remains elusive; however, there is convincing evidence that asthma is a highly inheritable disease (1, 30). To shed light on the genetic profiles of asthma and seek reasons attributable to the inconsistency of previous individual studies, we, in this meta-analysis, aimed to test the association of *ACE* gene I/D polymorphism with asthma risk, as well as with circulating ACE concentrations. Our genotype-disease and genotype–phenotype analyses showed that carriers of the mutant DD homozygote had a 50% increased risk of asthma and 3.13 U/L increased concentrations of circulating ACE relative to the wild II homozygote. Under the assumptions of the Mendelian randomization technique, it is expected that circulating ACE may be a causal risk factor for the development of asthma. The implication of circulating ACE in asthma is biologically plausible. There is evidence that ACE expressed in the lungs plays a key role in the pathogenesis of bronchial asthma. It is because ACE can mediate the proliferation of smooth vascular muscle cells (31), which affects aggregation and adhesion of platelets and monocytes and consequently leads to excessive bronchiectasis (32). The biological mechanism behind the causal implication of circulating ACE in asthma is not clear at present (33). It is reasonable to speculate that if involved, the mutation of I/D polymorphism can alter the expression of ACE in circulation or tissues, which triggers the development of asthma.

It is also worth noticing that according to our subsidiary analyses, differences in race, matched status, asthma diagnosis, sample size, and age might account for previously diverging findings of individual studies. Taking race as an example, we found that the susceptibility of *ACE* gene I/D polymorphism to asthma was race-dependent, with significance observed in Caucasians but not in East Asians, in agreement with the findings of previous studies (16, 18, 19, 21, 34). Indeed, asthma is a multifactorial disease to which genetic, environmental, and lifestyle-related factors contribute jointly (35). For feasibility reasons, it is recommended to construct a list of candidate genetic determinants for asthma in each racial group. Moreover, diagnostic criteria for asthma can also confound the association between *ACE* gene I/D polymorphism and asthma risk. In this meta-analysis, significance was observed in studies based on ATS and GINA criteria, whereas there was no observable significance in studies with self-reported asthma. To derive a reliable estimate, it is important to diagnose asthma formally. Differing from the observations of subsidiary analyses, we failed to reveal any statistical significance in meta-regression analyses, an alternative method to explore sources of between-study heterogeneity. It is of practical importance to bear in mind that meta-regression analyses, albeit enabling covariates in either continuous or categorical format to be regressed, do not have the methodological rigor of a properly designed study that is intended to test the effect of these covariates formally (36).

Another important finding is the obvious changes in circulating ACE between genotypes of *ACE* gene I/D polymorphism. Considering the fact that the I/D polymorphism is located in the 16th intron, it is unlikely to be functional at the transcription level. There is a possibility that this polymorphism is strongly linked to another functional locus in either the promoter or exon or 3′-untranslated region of the *ACE* gene that is responsible for the regulation of circulating ACE concentrations. Further genomic and functional explorations of the *ACE* gene are encouraged.

### Limitations

Several limitations should be acknowledged in this meta-analysis. First, this meta-analysis synthesized evidence from publications written in the English language, and selection bias cannot be excluded. Second, all included studies are cross-sectionally designed; however, causality was inferred by means of the Mendelian randomization technique. Third, only a few features were commonly provided by the majority of included studies, and it is expected that more features are needed to examine their potential confounding impact on between-study heterogeneity. Fourth, there was moderate evidence of publication bias, which might limit the generalizability of our findings.

### Conclusion

Taken together, we, for the first time, provided systematic evidence supporting the causal implication of high circulating ACE in the development of asthma by means of the Mendelian randomization technique. Further experimental studies are needed to determine the culprit genetic loci in the *ACE* gene that can simultaneously regulate circulating ACE concentrations and precipitate the onset and progression of asthma.

## Data availability statement

The original contributions presented in the study are included in the article/Supplementary material, further inquiries can be directed to the corresponding authors.

## Author contributions

QZ and WN planned and designed the study, and directed its implementation. QH and FY contributed to data acquisition. QH, YH, and BP conducted statistical analyses. QH and WN wrote and revised the manuscript. All authors read and approved the final manuscript prior to submission.

## Funding

This work was financially supported by the CAMS Innovation Fund for Medical Sciences (2021-12M-C&T-B-089) and the National Natural Science Foundation of China (Grant number: 81970042).

## Conflict of interest

The authors declare that the research was conducted in the absence of any commercial or financial relationships that could be construed as a potential conflict of interest.

## Publisher's note

All claims expressed in this article are solely those of the authors and do not necessarily represent those of their affiliated organizations, or those of the publisher, the editors and the reviewers. Any product that may be evaluated in this article, or claim that may be made by its manufacturer, is not guaranteed or endorsed by the publisher.
